# Streptomyces Bioactive Metabolites Prevent Liver Cancer through Apoptosis, Inhibiting Oxidative Stress and Inflammatory Markers in Diethylnitrosamine-Induced Hepatocellular Carcinoma

**DOI:** 10.3390/biomedicines11041054

**Published:** 2023-03-29

**Authors:** Sana M. Alhawsawi, Mohamed Mohany, Almohannad A. Baabbad, Nawaf D. Almoutiri, Saleh N. Maodaa, Esam M. Al-shaebi, Khadijah N. Yaseen, Mohammed A. M. Wadaan, Wael N. Hozzein

**Affiliations:** 1 Department of Zoology, College of Science, King Saud University, P.O. Box 55760, Riyadh 11451, Saudi Arabia; 2 Department of Pharmacology and Toxicology, College of Pharmacy, King Saud University, P.O. Box 55760, Riyadh 11451, Saudi Arabia

**Keywords:** anticancer activity, *Streptomyces*, hepatocellular carcinoma, apoptosis, oxidative stress, inflammation

## Abstract

A safe and effective treatment for liver cancer is still elusive despite all attempts. Biomolecules produced from natural products and their derivatives are potential sources of new anticancer medications. This study aimed to investigate the anticancer potential of a *Streptomyces* sp. bacterial extract against diethylnitrosamine (DEN)–induced liver cancer in Swiss albino mice and explore the underlying cellular and molecular mechanisms. The ethyl acetate extract of a *Streptomyces* sp. was screened for its potential anticancer activities against HepG-2 using the MTT assay, and the IC_50_ was also determined. Gas chromatography–mass spectrometric analysis was used to identify the chemical constituents of the *Streptomyces* extract. Mice were administered DEN at the age of 2 weeks, and from week 32 until week 36 (4 weeks), they received two doses of *Streptomyces* extract (25 and 50 mg/kg body weight) orally daily. The *Streptomyces* extract contains 29 different compounds, according to the GC-MS analysis. The rate of HepG-2 growth was dramatically reduced by the *Streptomyces* extract. In the mice model. *Streptomyces* extract considerably lessened the negative effects of DEN on liver functions at both doses. Alpha-fetoprotein (AFP) levels were significantly (*p* < 0.001) decreased, and P53 mRNA expression was increased, both of which were signs that *Streptomyces* extract was suppressing carcinogenesis. This anticancer effect was also supported by histological analysis. *Streptomyces* extract therapy additionally stopped DEN-induced alterations in hepatic oxidative stress and enhanced antioxidant activity. Additionally, *Streptomyces* extract reduced DEN-induced inflammation, as shown by the decline in interleukin-1 beta (IL-1β) and tumor necrosis factor-alpha (TNF-α) levels. Additionally, the *Streptomyces* extract administration dramatically boosted Bax and caspase-3 levels while decreasing Bcl-2 expressions in the liver according to the Immunohistochemistry examination. In summary, *Streptomyces* extract is reported here as a potent chemopreventive agent against hepatocellular carcinoma through multiple mechanisms, including inhibiting oxidative stress, cell apoptosis, and inflammation.

## 1. Introduction

Liver cancer is one of the most serious health problems now affecting the world. It is the second-most lethal type of cancer in the world, with a male-to-female ratio of 2.4:1.1 [1]. Hepatocellular carcinoma (HCC) accounts for around 90% of primary liver cancer [2]. HCC is a malignancy brought on by inflammation that results in a malignant neoplasm of the hepatocytes [3]. Hepatitis B virus (HBV), hepatitis C virus (HCV), frequent alcohol use, and non-alcoholic fatty liver disease are a few of the factors that might lead to the development of HCC [4]. Hepatic fibrosis in its advanced stages, smoking, inorganic arsenic in drinking water, aflatoxins, and iron buildup are all known key risk factors for the development of HCC as well [5].

Experimental mice models are being utilized more frequently in HCC studies to investigate the pathogenesis of the illness and to evaluate potential new treatments [6]. Diethylnitrosamine (DEN) is the most often used chemical to generate liver cancer in mice and has been used in the bulk of preclinical studies for some years [7]. When injected into young mice, DEN targets the liver, where it is biologically transformed into alkylating agents that can create mutagenic DNA adducts by centrilobular hepatocytes [8]. Additionally, there is proof that inflammation plays a role in the development of hepatocarcinogenesis brought on by DEN [9]. In addition to being a genotoxic substance, DEN is also hepatotoxic and causes necrotic cell death. This damage sets off an inflammatory response that increases the expression of mitogens such as interleukin-6 (IL-6), which encourages the proliferation of remaining hepatocytes as a form of reparative action [10].

Apoptosis is mediated by intrinsic and extrinsic signaling pathways, which can be triggered by a range of events, including cellular stress and DNA damage [11]. Apoptosis is dysregulated in several illnesses, including cancer, and the equilibrium between prosurvival and proapoptotic proteins regulates this process [12]. Uncontrolled, aberrant cell proliferation is a defining feature of cancer. Evasion of apoptosis is one of the characteristics of malignancies that promote tumor development and progression in addition to unchecked cell proliferation. The majority of anticancer medications work by directly triggering the apoptotic pathways in cancerous cells. Chemotherapeutic drugs such as Sorafenib are the most popular HCC treatments that have been developed over many years [13]. Chemotherapeutic drugs, however, typically target cancer rather than a particular type and frequently show a wide range of toxicities, either systemic and/or neural [14]. Therefore, a better therapeutic option or drug must be developed that specifically targets and inhibits the tumor-specific pathways in HCC. Natural products or their derivatives have drawn more attention in the treatment of cancer than other groups of anticancer medicines since they are abundant in nature and have little to no side effects.

Members of the genus *Streptomyces,* which belongs to the Streptomycetaceae family of the Actinobacteria (a group of Gram-positive bacteria), can produce several medicinal chemicals, including antibiotics, as well as the novel, naturally occurring secondary metabolites [15], with antitumor antioxidants, antibacterial, antifungal, antimicrobial, anti-hyperglycemic, anti-inflammatory, immunosuppressive activities [16]. According to allegations that *Streptomyces* species are no longer a significant biological source for new antibiotics, the rediscovery of known secondary metabolites from *Streptomyces* species has diverted scientists’ attention to the identification of uncommon actinobacteria for discovering new drugs. *Streptomyces* sp. is previously known to produce a variety of bioactive compounds with anticancer and antitumor characteristics, especially against human lung cancer [17], colorectal cancer [18], and prostate cancer [19]. These compounds showed cytotoxicity against malignant cells but did not harm healthy cells. To our knowledge, there is no information on the anticancer activity of a strain of *Streptomyces* sp. isolated from a habitat in Saudi Arabia against liver cancer. Therefore, the main goal of this study was to find a promising strategy to treat HCC using natural extract derived from *Streptomyces* isolated from the Saudi soil habitats and explore the underlying cellular and molecular mechanisms with focusing on the role of oxidative stress, inflammation and cell apoptosis.

## 2. Materials and Methods

### 2.1. Preparation of the Bacterial Extract

The pure *Streptomyces* strain, which was kindly provided by Prof. Wael N. Hozzein, was cultured in yeast extract-malt extract broth (ISP2) [20] and then incubated at 30 °C and 150 rpm for 2 days. Twenty 500 mL-conical flasks, each one containing 100 mL of starch casein broth [21], were then inoculated with 5 mL of the previously prepared inoculum. The flasks were incubated at 30 °C and 150 rpm for 5 days in a shaking incubator. After incubation, ethyl acetate was used for extraction of the metabolites produced by the *Streptomyces* strain under study.

### 2.2. Gas Chromatography–Mass Spectrometry (GC-MS) Analysis for the Chemical Constituents of the Streptomyces Extract

The identification of the chemical composition of *Streptomyces* extract was determined by a coupled Agilent Technologies 7890B GC System combined with Agilent Technologies 5977A MSD. *Streptomyces* extract was dissolved in ethyl acetate; the GC-MS was performed on: DB- 5 ms column (30 m × 0.32 mm × 0.25 µm), He carrier gas, the column head pressure was 10 psi, the oven temperature was sustained initially at 50 °C for 1 min, and then the programmed temperature was raised at a rate of 5 °C/min from 50 to 280 °C. In the end, the temperature was kept at 280 °C for 10 min. The analysis for each chemical compound was based on its retention time relative to those of authentic samples and matching spectral peaks available with the published data.

### 2.3. Determination of Cell Viability by MTT Assay

The anticancer activities of the natural *Streptomyces* sp. extract were investigated against HepG-2 (DSM ACC-180), the human hepatocellular carcinoma cancer cell line using the MTT method. The HepG-2 cancer cell line was cultivated and propagated on DMEM high glucose medium supplemented with 10% FBS and 1% penicillin-streptomycin. Cells were seeded into 96-well cell culture plates at a density of 10 × 10⁴ cells per well in 200 µL aliquots of the medium. The untreated cells served as the control. The cells were grown in a 5% CO_2_ incubator at 37 °C and 90% relative humidity. Cells were treated with *Streptomyces* sp. extract for 24 h at a concentration of 1 mg/mL dissolved in methanol. The serial dilutions of the extract were tested in triplicates at different concentrations (0, 0.125, 0.25, 0.5, and 1 mg/mL) (Nemati et al., 2013). Then, cell viability was evaluated by the cytotoxicity MTT assay. After 24 h, 20 µL MTT reagent was added for 2 h, as described by Oka et al. (1992). The MTT test is based on the reduction of the MTT reagent (3-(4,5-dimethylthiazol-2-yl)2,5-diphenyltetrazolium bromide) by living cell dehydrogenases to the violet formazan product, and 200 µL/well of 1X isopropanol-HCL was added after the MTT reagent. The absorbance was measured at 595 nm using a microplate reader (Zenyth 200 ST, Biochrom, UK), and the inhibition of cell growth was calculated. The results have also been plotted to give the cytotoxicity activity curve for each extract, and the LC50 was calculated.

Cell viability was calculated using the following equation [22]:

Cell viability (%) = (O.D of treated sample)/(O.D of untreated sample) × 100%

### 2.4. Animals and Chemicals

Pregnant female Swiss albino mice were obtained from the Animal House, Zoology Department, College of Science, King Saud University. Animals were housed in stainless steel wire cages under pathogen-free conditions. Before the experiment, all mice were given 7 days to acclimate in polycarbonate cages in a well-ventilated environment. Standard laboratory settings (temperature of 23–24 °C, relative humidity of 50–60%, and a 12-h light/dark cycle) were used to sustain the animals, and they were provided with food and water ad libitum. DEN was purchased from Sigma-Aldrich, CAS No. 55-18-5 (St Louis, MO, USA).

### 2.5. General Experimental Procedures

A total of 60 male mice, obtained by mating male and female, were given an intraperitoneal (I.P.) injection of 25 mg kg^−1^ diethylnitrosamine (DEN) at the age of 2 weeks, as previously reported [23], and about 30 weeks later, liver tumors appeared. The mice were then left with their mothers to finish the nursing period. Each set of pups during nursing is assigned a distinct color for their tails to help with organizing. The females were not included once they reached the age of 4 weeks. Because the male gender is a risk factor for human HCC, male mice were used in the current study [24]. At 6 weeks old, mice were randomly assigned to 6 groups based on weight. In each group, there are 10 mice. Once every week, mice were weighed. These were the experimental groups: Group 1 served as the negative control group, receiving 25 mg kg^−1^ b.w. of normal saline orally for 4 weeks. Group 2 received 25 mg kg^−1^ b.w. of low *Streptomyces* sp. extract concentration orally for 4 weeks. Group 3 received 50 mg/kg b.w. of high *Streptomyces* sp. extract concentration orally for 4 weeks. Group 4: positive control group received only a single I.P. dose of DEN (25 mg/kg b.w) at 2 weeks of age. Group 5: mice received a single I.P. dose of DEN at the age of 2 weeks and the low *Streptomyces* sp. extract dose (25 mg/kg b.w.) orally for 4 weeks. Group 6: mice received a single I.P. dose of DEN at the age of 2 weeks and the high *Streptomyces* sp. extract dose (50 mg/kg b.w.) orally for 4 weeks. All of the mice in groups 4, 5 and 6 received a single i.p. injection of DEN (25 mg/kg body weight) at 2 weeks of age. This concentration of *Streptomyces* sp. was established by prior publications [25], and treatment with the extract of *Streptomyces* sp. started in week 32 and lasted for 4 weeks until week 36.

### 2.6. The Toxicity Test for the Natural Extract

Ten mice were given varied concentrations of the extract, ranging from 25 mg/kg body weight to 100 mg/kg body weight, to determine the toxicity of the extract. The toxic symptoms and mortality were noted during the course of the next 7 days.

### 2.7. Sampling and Biochemical Analysis

After the 36th week, 10% ketamine (Hikma Pharmaceuticals, Jordan, 100 mg/kg) and 2% xylazine (Laboratories Calier, Spain, 10 mg/kg) were combined intraperitoneally to anesthetize all the animals. The blood was collected from the jugular vein, then allowed to coagulate at room temperature, and centrifuged at 3000 rpm for 30 min. The serum was quickly removed and stored at −20 °C for subsequent biochemical analyses. After dissection of the animals, the liver tissues were immediately excised, washed in saline, and fixed in 10% neutral buffer formalin for histopathological examination. Liver samples were homogenized in phosphate-buffered saline (PBS) solution and centrifuged at 3000 rpm for 10 min, and the clear supernatants were kept in a deep freezer at −20 °C for further analyses. The remaining liver tissues were stored at −80 °C. The biochemical investigations, oxidative stress, and antioxidant markers in hepatic tissues were assessed using the Jenway 6300 spectrophotometer. Serum alanine aminotransferase (ALT) and aspartate aminotransferase (AST) were determined using commercial kits (Bio diagnostics, Egypt, CAT No. AL 10 31 (45), 10 61 (45), respectively).

### 2.8. Oxidative Stress and Antioxidant Markers in Liver Homogenate

As per the manufacturer’s instructions, Bradford reagent was used to calculate the total protein in each liver sample. Malondialdehyde (MDA), which is produced as a byproduct of lipid peroxidation, is a measurement of the degree of oxidative stress. Using biodiagnostic kits, and diagnostic and research reagents, in Egypt, we assessed the hepatic tissue contents of GSH (CAT No. GR 25 11), malondialdehyde (MDA, CAT No. MD 25 29), GST (CAT No. GT 25 19), and GPx (CAT No. GP 25 24) in liver homogenate according to the manufacturer’s guidelines.

### 2.9. Analysis of Gene Expression: Quantitative PCR (RT-qPCR)

Total RNA was extracted from the liver harvested using TRizol^®^ Reagent (Invitrogen, Paisley, UK) according to standard procedures. The quality and quantity of the purified RNA were determined by a Qubit^®^ 2.0 fluorometer using the Qubit RNA assay kit. To eliminate any contaminating genomic DNA, a gDNA wipeout reaction was undertaken in a wipeout buffer at 42 °C for 2 min. CDNA was synthesized from total RNA using the QuantiTect Reverse Transcription Kit (QIAGEN, QuantiTect^®^, Germany), according to the manufacturer’s instructions. Gene expression of target genes (AFP, IL-1β, TNF-α, and P53) was determined by QuantiTect SYBER-GREEN PCR kit (QIAGEN, Germany). Amplification reactions of the target genes were performed in 96-well plates containing SYBR^®^ Green Master Mix (QIAGEN, Germany), cDNA, and the specific oligonucleotide primers shown in Table 1. Expression was normalized to GAPDH gene expression, which was used as an internal housekeeping control. Raw data were analyzed using the Rotor-Gene cycler software version 2.3 to calculate the threshold cycle using the second derivative maximum. The obtained data were analyzed using the 2−∆∆Ct method.

### 2.10. Histopathological Investigations

Harvested liver tissues were promptly fixed in 10% neutral buffer formalin in PBS, followed by a wash in PBS, a series of alcohol dehydration, and fresh paraffin embedding. Microtome slices (5 µm thick) were prepared by cutting them, soaking them in warm distilled water (~40 °C), then picking them up on slides of glass. The slides were stained with hematoxylin and eosin after being incubated on a vertical rack for a whole night at 62 °C. At 100× magnification, slides were captured on a high-resolution digital camera and examined by an experienced pathologist using a light microscope, the Nikon Eclipse E600. To evaluate the degree of liver injury, an injury grading score (Grade 0–4) based on the severity of lesions in the liver parenchyma was carried out, as previously reported [26].

### 2.11. Immunohistochemistry Procedure of Paraffin Sections

Immunohistochemistry was carried out on formalin-fixed, 3 µm paraffin-embedded liver sections mounted on positively charged slides using the avidin-biotin-peroxidase complex (ABC) technique. Sections were treated with the anti-caspase-3 rabbit pAb antibody, ABclonal Cat# A11953; the anti-Bcl-2 rabbit polyclonal antibody, ABclonal Cat# A16776; and the anti-Bax rabbit polyclonal antibody, Bioss ANTIBDIES Cat# bs-0127R after blocking for 30 min with Rodent Block M. The previously indicated antibodies were applied to the sections of each group before the ABC technique reagents (Vectastain ABC-HRP kit, Vector labs) were added. Marker expression was peroxidase-labeled and diaminobenzidine-colored to detect antigen-antibody complexes (DAB, manufactured by Sigma). Slides were mounted with Eukitt^®^ mounting media and counterstained with Mayer’s hematoxylin. By leaving out the primary antibody on neighboring sections, negative controls were obtained. Light microscopy was used to examine the sections with a Leica microscope (CH9435 Hee56rbrugg) (Leica Microsystems, Switzerland). For each section, at least 20 separate, non-overlapping fields were looked at. The area % of immunohistochemically positive structures was quantified using ImageJ (NIH, Bethesda, MD, USA).

### 2.12. Statistical Analysis

The data were presented as means ± standard error of the mean (SEM). To determine statistical significance among the experimental groups using GraphPad 8, a one-way ANOVA followed by a Tukey’s test was used. A *p*-value less than 0.05 was considered statistically significant.

## 3. Results

### 3.1. Gas Chromatography–Mass Spectrometry (GC–MS) Analysis of Streptomyces sp. (A16) Extract

**Figure 1:**
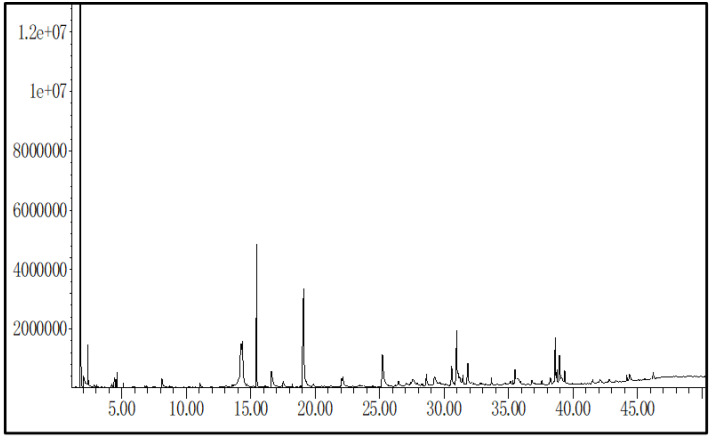
Chromatogram of *Streptomyces* sp. (A16) compounds in extract Ethyl acetate extract. The major compounds were Benzeneacetamide, trans-Cinnamaldehyde, Pyrrole-2-carboxamide and Cinnamamide.

By using GC-MS analysis, the chemical composition of the extract of *Streptomyces* sp. (A16) was identified. The data demonstrate the presence of 29 chemical components. Table 2 and Figure 1 show the concentrations of the primary constituents greater than 1% of the total composition. The chemicals’ mass spectra were analyzed and identified by comparison with the standard library data sources.

### 3.2. Antiproliferative Effects of Streptomyces sp. (A16) HepG2 Cells Viability

**Figure 2:**
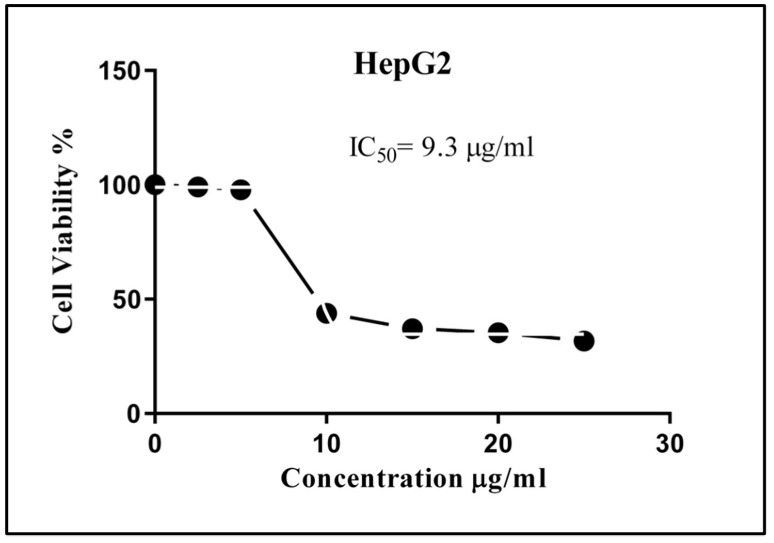
The percentage cytotoxicity on the HepG2 cell line was determined by MTT assay. Cells were cultured in 96-well plates and then treated with different concentration doses of *Streptomyces* sp. (A16) extract. The data are presented as mean  ±  SEM of three independent experiments and statistically analyzed by the unpaired *t*-test. The IC50 value was determined.

By using the MTT test, the aqueous methanol extract of *Streptomyces* sp. (A16) was evaluated for its ability to suppress the growth of HpG2 human liver cancer cell lines. The anti-proliferation impact of the microbial extract was demonstrated by the MTT test findings. The experiment was run three more times at different doses (0, 2.5, 5, 10, 15, 20, 25 g/mg). For 24 h, the *Streptomyces* sp. (A16) extract was incubated with the HpG2 cell line. (Figure 2) The control groups are represented by 0 g/mg (only methanol was added to the HpG2 cell line). The IC_50_ values (9.3 g/mL) for *Streptomyces* sp. (A16) extract on HpG2 human cell lines provide evidence of its effects.

### 3.3. Acute Toxicity Test

Within 24 h of the extract’s oral treatment, no dead mice were seen. No toxicity sign effects, such as paw licking, hair erection, decrease in feeding activity, and an increase in respiration rate, were observed in the mice’s behavior. At a dosage of 60 mg/kg body weight, *Streptomyces* extract started to show signs of toxicity.

### 3.4. The Impact of Streptomyces sp. (A16) Extract on the Architecture, Weight, and Serum AST and ALT Levels of the Liver Tissues in DEN-Induced Hepatocarcinogenesis

**Figure 3:**
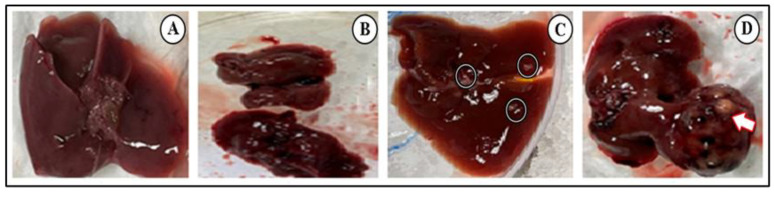
Representative images of mice livers showing tumor progression over time, control group (**A**), DEN-treated group after 3 months (**B**), DEN-treated group after 6 months showing unequal sized gray nodules, which was a signal for HCC formation (**C**), DEN-treated group after 9 months showing showed liver swelling, cirrhosis, unequal size, white tumor nodules (arrow) (**D**).

The formation of HCC was investigated using macro-alterations in the liver tissues. The liver tissue of the normal control group revealed no clear macroscopic alterations (Figure 3A), but six months later, the DEN-treated group exhibited gray nodules of variable sizes, which was a sign of the development of HCC. Small quantities and different sizes of nodules were displayed (Figure 3C). However, the same group experienced an increase in tumor size and number nine months later. Nine months following the injection of DEN, the group showed clear signs of liver enlargement, cirrhosis, and unequally sized gray and white cancer nodules (Figure 3D).

The average final body weight of mice, including their liver weight, is shown in Table 2. The body weight of the mice group that received only DEN was significantly decreased in comparison to the normal control group (41 ± 0.9 vs. 44.4 ± 1.6, *p* < 0.001). In comparison to the mice group that received only DEN, mice treated with either DEN + A16 (L) or DEN + A16 (H) *Streptomyces* sp. had significantly (*p* < 0.01 or *p* < 0.001, respectively) higher body weights. Additionally, the liver weight of the mice that received only DEN was significantly higher than the liver weight of the normal control group (3.3 ± 0.08 vs. 2.3 ± 0.06, *p* < 0.01). The average liver weight was significantly lower in the mice group given either a low dose of 25 mg/kg b.w. of *Streptomyces* sp. extract or a high dose of 50 mg/kg b.w. of *Streptomyces* sp. extract (*p* < 0.01 or *p* < 0.001, respectively) after receiving DEN injections. There were no adverse effects of *Streptomyces* sp. extract (A16) on mice body and liver weight in groups that received the bacterial extract only.

ALT and AST blood tests are usually used in the laboratory as sensitive parameters to examine the presence of a liver injury. In the current study, we investigated the effects of *Streptomyces* sp. (A16) extract, which was administrated orally for 4 weeks, on the activities of ALT and AST in mice (Table 3). A significant increase in ALT (37.35 ± 0.5 U/L vs. 125.5 ± 1.5 U/L, *p* < 0.001) and AST (106.4 ± 1.11 U/L vs. 227.9 ± 5.74 U/L, *p* < 0.001) activities was detected in mice that received only intraperitoneal administration of DEN compared to the normal control group. In comparison to mice in the group that received only DEN, a significant reduction (*p* < 0.001) in ALT and AST levels was observed in the group that received (DEN+ A16 (L) and (DEN + A16 (H) doses of *Streptomyces* sp. (A16) extract. The levels of ALT in groups (DEN+ A16 (L) and (DEN + A16 (H) were 48.76 ± 0.53 and 41.00 ± 1.11 U/L, respectively, whereas AST values in these two groups were 116.7 ± 0.88 and 109.0 ± 2.17 U/L, respectively.

### 3.5. Effects of Streptomyces sp. Extract on Oxidative Stress and Antioxidant Defense Markers in DEN-Treated Mice

**Figure 4:**
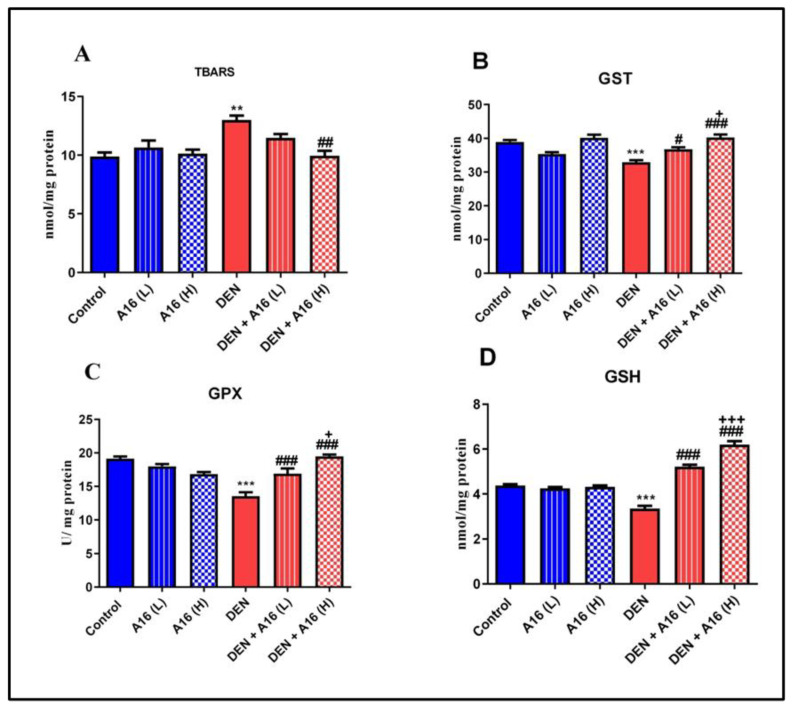
Effects *of Streptomyces* sp. (A16) extract on antioxidant enzyme activities and lipid peroxidation in mice liver samples following DEN injection, including TBARS (**A**), GST (**B**), GPx (**C**), and GSH (**D**). Data are presented as mean ± SEM (*n* = 6) and statistically analyzed by one-way ANOVA followed by the Tukey–Kramer post hoc test. ** *p* < 0.01, *** *p* < 0.001 were significant compared with the control group, ^#^ *p* < 0.05, ^##^ *p* < 0.01, ^###^ *p* < 0.001 were significant compared with DEN-treated mice and ^+^ *p* < 0.05, ^+++^ *p* < 0.001 were significant compared with DEN+ A16 (L)-treated mice. Abbreviations: TBARS, thiobarbituric acid reaction substances; GST, glutathione-S-transferase; GPx, glutathione peroxidase; GSH, glutathione.

Hepatic oxidative stress was observed in DEN-treated mice, which was related to higher levels of TBARs than in control mice (DEN, 12.9 nmol/mg vs. Control, 9.8 nmol/mg; *p* < 0.01). On the other hand, after 4 weeks of treatment with a high dose of *Streptomyces* sp. (A16) extract, the elevated TBARs levels brought on by the DEN injection were markedly reduced (DEN+ A16 (H), 9.9 nmol/mg vs. DEN, 12.9 nmol/mg; *p* < 0.01) compared with DEN-treated mice (Figure 4A). Additionally, it was shown that the mice treated with DEN had significantly (*p* < 0.001) lower levels of GST, GPx, and GSH than the mice in the normal control group. Treatment with both dosages of *Streptomyces* sp. (A16) extract significantly decreased (*p* < 0.05, *p* < 0.001) the effects of the DEN injection on GST, GPx, and GSH levels, with DEN + A16 (H) being more efficient than DEN + A16 (L) in enhancing antioxidant parameters (Figure 4B–D).

### 3.6. Effects of Streptomyces sp.(A16) Extract on mRNA Expressions of AFP, IL-1β, TNF-α and P53 in DEN-Induced Hepatocarcinogenesis

**Figure 5:**
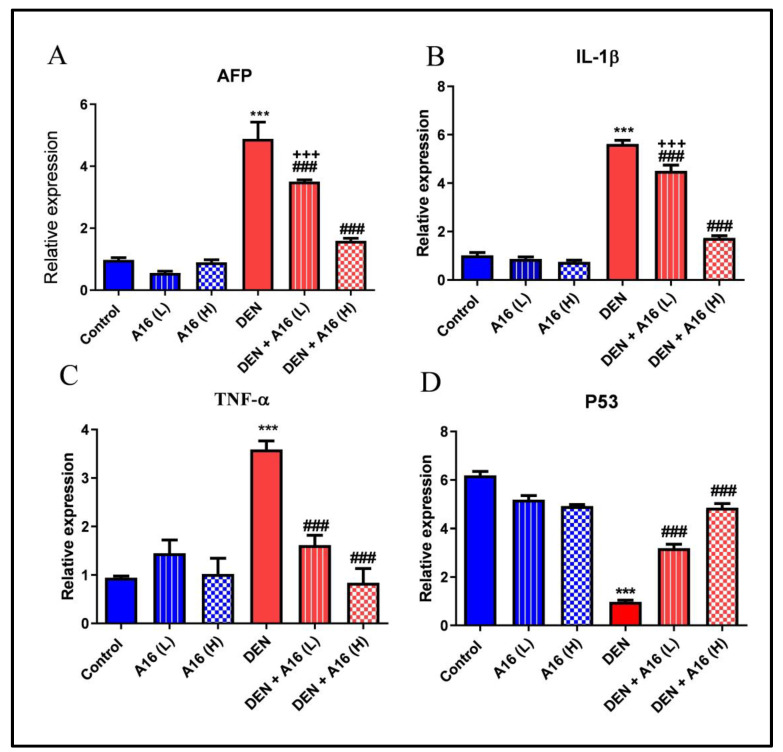
Assessment of mRNA expressions of AFP (**A**), IL-1β (**B**), TNF-α (**C**) and P53 (**D**) genes in hepatic tissues following treatment with *Streptomyces* sp. (A16) extract in DEN-administered mice. Data are presented as mean ± SEM (*n* = 6) and statistically analyzed by one-way ANOVA followed by the Tukey–Kramer post hoc test. *** *p* < 0.001 were significant compared with control group, ^###^ *p* < 0.001 were significant compared with DEN-treated mice and ^+++^ *p* < 0.001 was significant compared with DEN + A16 (L)-treated mice. Abbreviations: AFP, alpha-fetoprotein; IL-1β, interleukin-1β; TNF-α, tumor necrosis factor-α.

All experimental groups’ liver tissues underwent gene expression analysis using qPCR to measure the levels of AFP, TNF-α, IL-1β, and P53 expressions, and values were standardized to the mRNA expression of hepatic GAPDH (Figure 5). Data on the expression of earlier genes in mice treated exclusively at 2 weeks of age with DEN showed a significant up-regulation (*p* < 0.001) compared to untreated control mice.

In DEN-treated mice, the expression of AFP was significantly higher by 4.4-fold compared to the normal control group (4.886 ± 0.53 vs. 0.9767 ± 0.07, *p* < 0.001). The expression level of AFP was significantly (*p* < 0.001) down-regulated in the mice treated with low or high doses of *Streptomyces* sp. (A16) extract and DEN by 3.5-fold and 1.5-fold respectively, compared to the group that received DEN only, and the high dose of *Streptomyces* sp. (A16) extract was more effective (*p* < 0.001) than the low dose of *Streptomyces* sp. (A16) extract in reducing AFP expression. Similarly, the expression level of IL-1β was elevated (*p* < 0.001) by 5.6-fold in the DEN-treated group compared to the control group, while these expressions were reduced 4.5-fold and 1.7-fold in the groups treated with low or high doses of *Streptomyces* sp. (A16) extract compared to the group that received DEN alone (Figure 5B). The expression level of TNF-α was also higher by 3.5-fold in DEN-treated mice compared to the control group (DEN, 3.58 ± 0.17 vs. control, 0.94 ± 0.03, *p* < 0.001) (Figure 5C). In contrast, TNF-α expression was significantly reduced in mice treated with low or high doses of *Streptomyces* sp. (A16) extract (1.6-fold and 0.8-fold, respectively) compared to the group that received DEN alone. The fold of gene expression change in the P53 gene was significantly lower by 6.1 (*p* < 0.001) in DEN-treated mice compared to the control group, while the P53 expression level was significantly augmented (*p* < 0.001) in groups treated with low or high doses of *Streptomyces* sp. (A16) extract, respectively (Figure 5D).

### 3.7. Effects of Streptomyces sp.(A16) Extract on Pathological Changes in DEN-Induced Hepatocarcinogenesis

**Figure 6:**
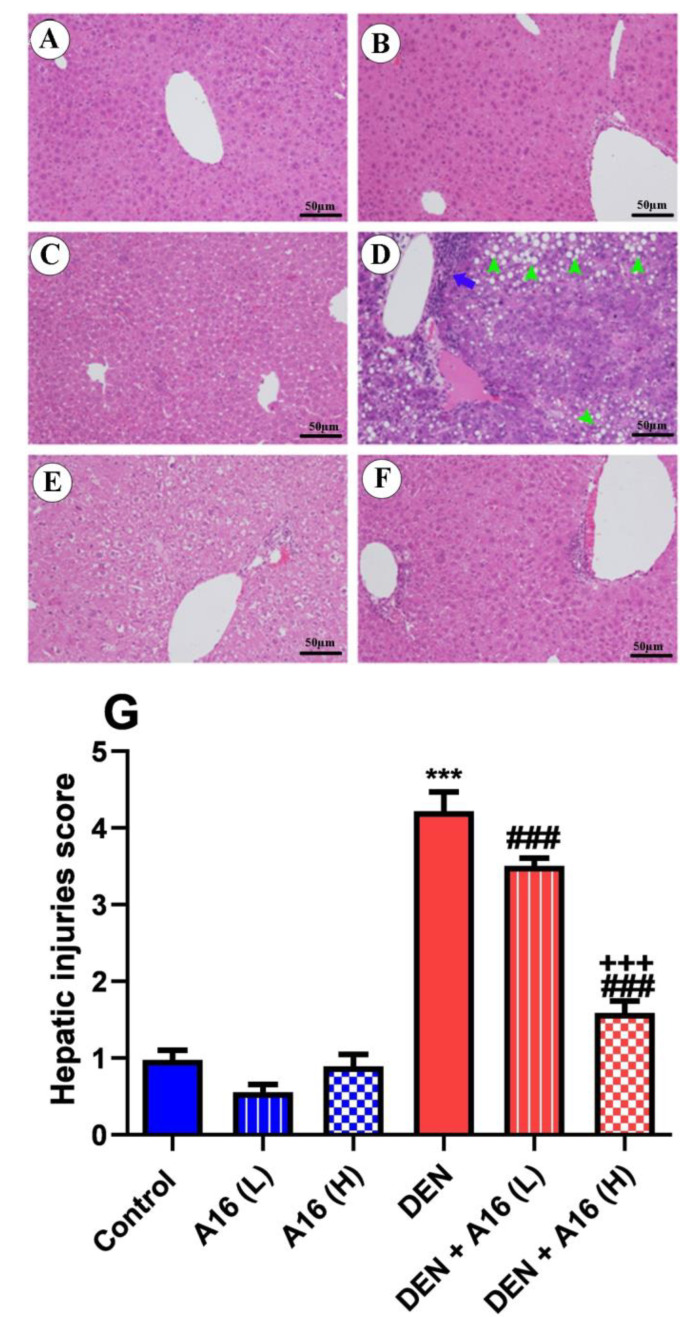
Histological examination of hepatic tissues following treatment with *Streptomyces* sp. (A16) extract in DEN-administered mice. (H&E, scale bar = 50 µm). (**A**) Liver tissue from the control group displaying typical hepatic cells. (**B**,**C**) Liver sections taken from mice that received a low dose (25 mg/kg body weight) or a high dose (50 mg/kg body weight) of *Streptomyces* sp. (A16) extract showing normal hepatic cells. (**D**) Liver tissue from the DEN-treated group exhibited abnormal liver tissue characteristics, such as the destruction of the hepatic lobules’ structural integrity, necrotic hepatic cells with multiple inflammatory infiltrations (blue arrow), fatty change (steatosis) (green head arrows), variable nuclei size, loss of cell membrane, and absence of the hepatic sinusoids’ capillaries. Along with the Kupffer cell hyperplasia, other changes included hyaline changes, bile duct dilatation, and hepatic artery and portal vein enlargement. (**E**,**F**) Liver tissue from the DEN+ A16 (L) and DEN + A16 (H) groups showed a significant improvement in liver architecture in the form of decreased hepatic necrosis, fewer inflammatory cells, reduced steatosis, and modest cytoplasmic vacuolization of hepatocytes. (**G**) Semiquantitative histological scoring of the liver injuries. Data are presented as mean ± SEM (*n* = 5) and statistically analyzed by the Kruskal-Wallis test and Dunn’s multiple comparison post hoc test. *** *p* < 0.001 were significant compared with the control group, ^###^ *p* < 0.001 were significant compared with DEN-treated mice, and ^+++^ *p* < 0.001 were significant compared with DEN+ A16 (L)-treated mice.

**Figure 7:**
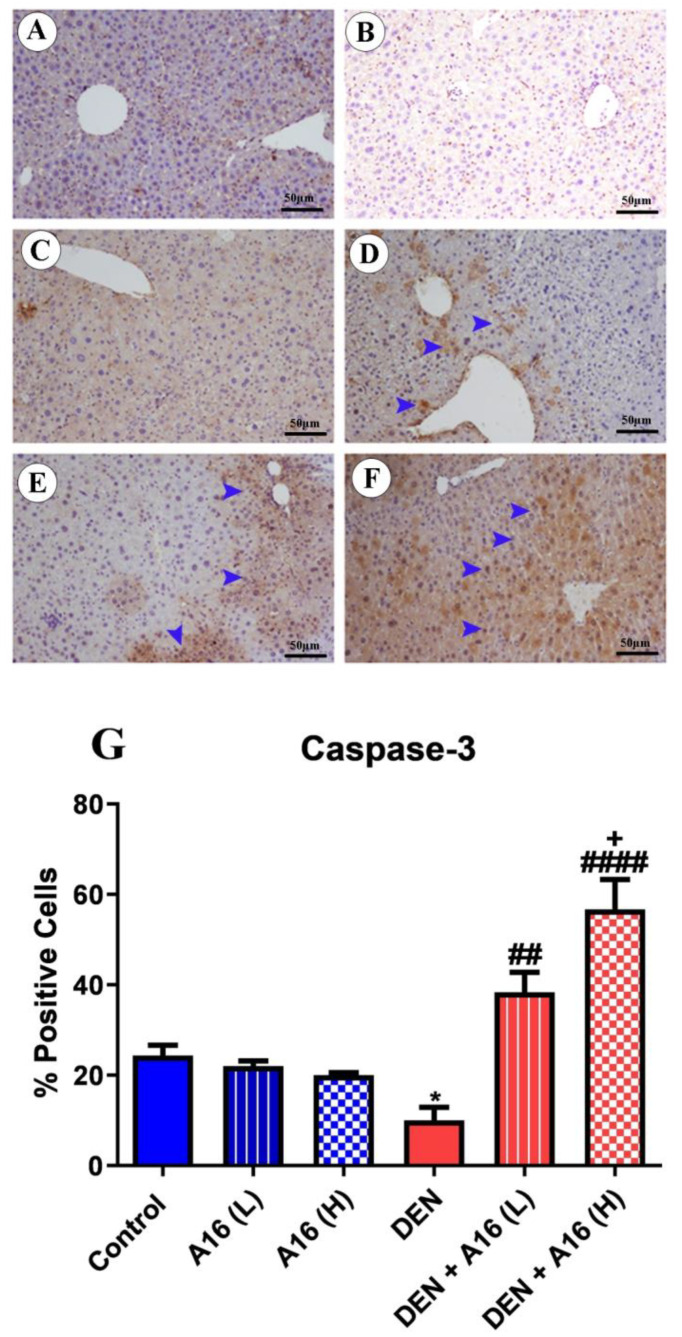
Liver caspase-3 immunohistochemistry. Photomicrographs of sections of liver samples taken from (**A**) the control. (**B**,**C**) Liver sections taken from mice that received a low dose (25 mg/kg body weight) and a high dose (50 mg/kg body weight) of *Streptomyces* sp. (A16) extract, respectively. (**D**) DEN-treated group. (**E**,**F**) Liver tissue from the DEN+ A16 (L) and DEN + A16 (H) *Streptomyces* sp. extract. The area percent of positive caspase-3 immunoreactivity (head arrows) was quantified (**G**). Values are presented as mean ± SEM (*n* = 3) and statistically analyzed by one-way ANOVA followed by the Tukey–Kramer post hoc test. * *p* < 0.05 versus control group, ^##^ *p* < 0.01, ^####^ *p* < 0.0001 versus DEN-treated mice and ^+^ *p* < 0.05 versus DEN+ A16 (L)-treated mice.

All sections of mice livers were stained with hematoxylin and eosin (H&E) for histopathological examination to study the effect of oral administration of *Streptomyces* sp. (A16) extract for 4 weeks after receiving a single I.P. DEN injection (Figure 6). The section of liver tissue of the normal control mice showed the normal appearance of hepatic lobules. The hepatocytes were arranged radiating outward from a central vein. Sinusoid capillaries separatie the plates of hepatocytes. Kupffer cells are located adjacent to the liver sinusoids (Figure 6A). Mice that received solely *Streptomyces* sp. (A16) extract at low doses of 25 mg/kg b.w. and high dosages of 50 mg/kg b.w. were found to have a similar normal liver architecture in sections. The extract has no negative effects on the liver tissues (Figure 6B,C). The liver sections of the DEN-treated group showed abnormal liver tissue characteristics, such as loss of hepatic lobule architecture, inflammation, necrosis, apoptosis, fatty change (steatosis), variable nuclei size, loss of cell membrane, and absence of hepatic sinusoids capillaries. Hyaline altered in addition to the Kupffer cells, showing signs of hyperplasia. Lastly, it has been demonstrated that connective tissue can be ruptured (Figure 7D). Co-administration of low or high doses of *Streptomyces* sp. (A16) extract after DEN injection attenuated these histopathological changes (Figure 6E,F). The reduction in severe liver lesions in the group treated with a low dose of *Streptomyces* sp. (A16) extract was observed. In the mice sections of the group of DEN + high dose of the *Streptomyces* sp. (A16) extract, distortion of hepatocytes was reduced as well as rejuvenation of hepatic architecture. Less fatty change, the least pleomorphism, and re-establishment of hepatocyte morphology have been recorded in comparison to the HCC control group. These histological alterations were lessened by co-administration of low or high doses of *Streptomyces* sp. (A16) extract following DEN injection (Figure 6E,F). It was noticed that the group given a low dose of *Streptomyces* sp. (A16) extract had fewer serious liver lesions. The deformation of the hepatocytes as well as the regeneration of the hepatic architecture were reduced in the mouse sections of the group of DEN + high dose of the *Streptomyces* sp. (A16) extract. In comparison to the HCC control group, less fatty alteration, less pleomorphism, and re-establishment of hepatocyte morphology have been observed. The semiquantitative histological analysis findings showed that the liver slides from the DEN-treated group had significantly (*p* < 0.001) more damage than those from the healthy control group. Intriguingly, most of the hepatic histological changes brought on by DEN injection were significantly attenuated (*p* < 0.001) in the treated groups after 4 weeks of treatment with low or high doses of *Streptomyces* sp. (A16) extract. Treatment with the high dose of *Streptomyces* sp. (A16) extract was more effective in reducing the induced hepatic lesions (Figure 6G).

### 3.8. Immunohistochemical Analysis of Bcl2, Bax, and Caspase-3 after Streptomyces sp.(A16) Extract Treatment of DEN-Induced Hepatocarcinogenesis

**Figure 8:**
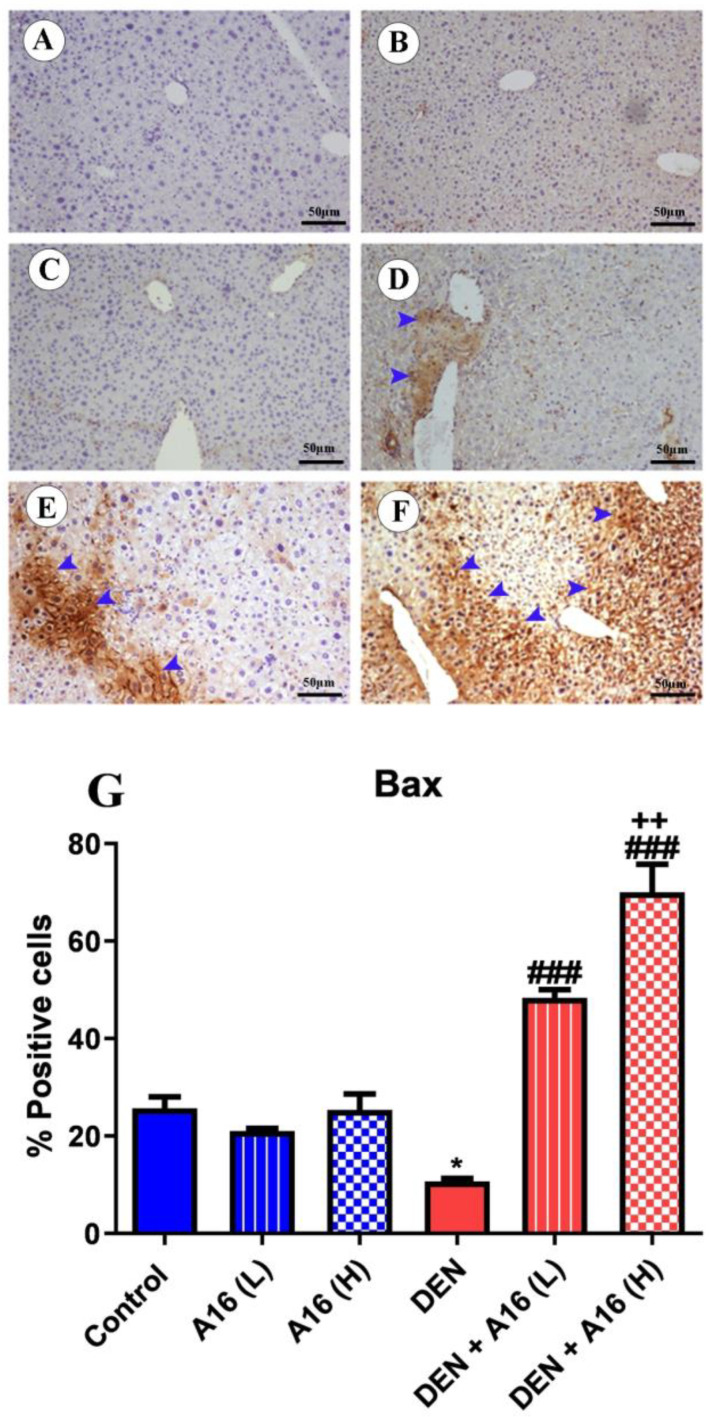
Liver Bax immunohistochemistry. Photomicrographs of sections of liver samples from the following groups: (**A**) Control group, (**B**,**C**) liver sections taken from mice that were given a low dose (25 mg/kg body weight) and a high dose (50 mg/kg body weight) of *Streptomyces* sp. (A16) extract respectively, (**D**) DEN-treated group, (**E**,**F**) liver tissue from the DEN+ A16 (L) and DEN + A16 (H) *Streptomyces* sp. extract. The area percent of positive Bax immunoreactivity (head arrows) was quantified (**G**). Data are expressed as mean ± SEM (*n* = 3) and statistically analyzed by one-way ANOVA followed by the Tukey–Kramer post hoc test. * *p* < 0.05 versus control group, ^###^ *p* < 0.001 versus DEN-treated mice and ^++^ *p* < 0.01 versus DEN+ A16 (L)-treated mice.

We conducted an immunohistochemistry study for the apoptosis markers to clarify the potential proapoptotic effect of the *Streptomyces* sp.(A16) extract. According to the immunohistochemical analysis in the present study, caspase-3 expression in DEN-treated mice significantly decreased (*p* < 0.05) and was significantly augmented by *Streptomyces* sp.(A16) extract in a dose-dependent manner, demonstrating the capacity of *Streptomyces* sp.(A16) to induce apoptosis of cancers liver cells (Figure 7). The expression of Bax in the hepatic tissue of mice treated with DEN decreased significantly (*p* < 0.05) when compared to samples from control animals. These modifications were dramatically increased (*p* < 0.001) by administering low or high dosages of *Streptomyces* sp. (A16) extract, which led to positive foci Bax responses. Higher positive cells in Bax expression were seen in DEN-treated mice treated with high doses of *Streptomyces* sp. (A16) extract compared to animals treated with low doses of *Streptomyces* sp. (A16) extract (*p* < 0.01) (Figure 8).

**Figure 9:**
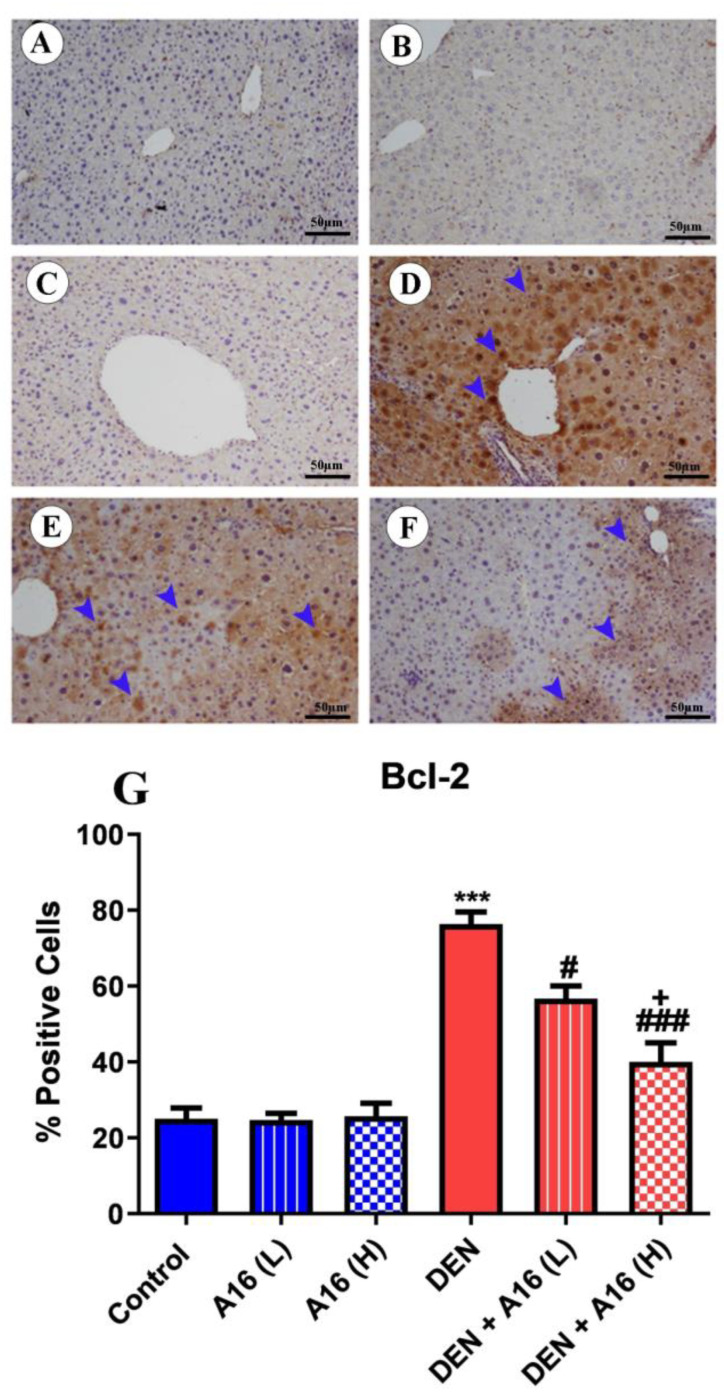
Liver Bcl-2 immunohistochemistry. Photomicrographs of sections of liver samples from the following groups: (**A**) Control group, (**B**,**C**) liver sections taken from mice that were given a low dose (25 mg/kg body weight) and a high dose (50 mg/kg body weight) of *Streptomyces* sp. (A16) extract, respectively, (**D**) DEN-treated group, (**E**,**F**) liver tissue from the DEN+ A16 (L) and DEN + A16 (H) *Streptomyces* sp. extract. The area percent of positive Bcl-2 immunoreactivity (head arrows) was quantified (**G**). Data are expressed as mean ± SEM (*n* = 3) and statistically analyzed by one-way ANOVA followed by the Tukey–Kramer post hoc test. *** *p* < 0.001 versus control group, ^#^ *p* < 0.05, ^###^ *p* < 0.001 versus DEN-treated mice and ^+^ *p* < 0.05 versus DEN+ A16 (L)-treated mice.

On the other hand, hepatic sections from the DEN-treated group showed considerably more Bcl-2 expression than those from control animals (*p* < 0.001). Although the expression of Bcl-2 was significantly reduced following treatment with low or high doses of *Streptomyces* sp. (A16) extract (*p* < 0.05, *p* < 0.001, respectively), the high dose effect of *Streptomyces* sp. (A16) was superior to that of the low-dose treatment (*p* < 0.05) (Figure 9). The current immunohistochemistry data indicate that *Streptomyces* sp. (A16) extract could cause malignant liver cells to undergo apoptosis.

## 4. Discussion

This study aimed to identify a promising method for treating HCC by using newly isolated actinobacteria from Saudi soil habitats. We used HepG2 cell lines, which are frequently used as human HCC lines, to further analyze the anti-HCC properties of *Streptomyces* sp. (A16) extract. *Streptomyces* sp. (A16) extract suppressed the growth of the HepG2 cell line, as demonstrated by the MTT assay results, demonstrating a broad spectrum of *Streptomyces* sp. (A16) extract inhibitory effects on human HCC cell growth, as previously reported [27,28]. As a result of these results, we decided to test *Streptomyces* sp. (A16) extract on an HCC animal model.

In the present study, we found that the body weight of the mice group that received only DEN decreased significantly in comparison to the normal control group. According to earlier research, liver malignancy leads to a reduction in the body, and some of the solid cancer characteristics cause obligatory weight loss [29]. Regarding increasing the relative liver weight in cancer, the group that only received DEN in the current study had a higher liver weight than the other groups. A rise in the amount of water in the liver may be the cause of the increased liver weight. An increase in passive reserve materials, such as fat and glycogen, is thought to reflect the work the organ has performed. Therefore, an increase in liver weight could result from the identical activity being undertaken in this organ [30]. Additionally, the injection of DEN into mice in the current study at the age of two weeks resulted in hepatic injury and, as a result, changes in liver functions, as seen by the elevated levels of AST and ALT. Increased serum levels of AST and ALT in response to DEN treatment have been shown in numerous investigations [31,32]; these effects, which are linked to the development of HCC, may have been caused by leakage from damaged or necrotic cells as well as rising cell membranes permeability. Liver function indices significantly improved when *Streptomyces* sp. (A16) extract was administered to DEN-treated mice. The antioxidant content of the extract used in these treatments may be the cause of their hepatoprotective benefits.

Oxidative stress can damage DNA or alter protein expression, which can result in a variety of illnesses, including cancer. Because oxidative stress increases oxidative damage to DNA in the hepatocytes, it may be one of the risk factors for the development of HCC [33]. According to our findings, DEN-treated animals produced more hepatic TBARs and had lower levels of GPx, GST, and GSH, which indicated a degree of cell damage and an overt oxidative stress condition. Healthy cells frequently use antioxidant defense mechanisms, including GST, GPx, and GSH, to fend against ROS. However, this antioxidant defense system was severely halted by the injection of DEN [34]. The major finding in this study was that *Streptomyces* sp. (A16) extract significantly decreased liver TBARs, enhanced GPx and GST activity, and raised hepatic GSH levels in DEN-treated mice. The potential of *Streptomyces* sp. (A16) extract to shield the liver from oxidative stress is demonstrated by decreased TBAR levels, improvements in liver GSH content, and antioxidant enzyme activities [35]. Actinomycetes extracts have been demonstrated to improve liver structure and function after exposure to carcinogens and to restore antioxidant enzymes [16]. The bioactive compound 9-Octadecenamide, which has been demonstrated to have anti-inflammatory and antioxidant actions [36], is present in *Streptomyces* sp. (A16) extract, which may be in charge of the recovery of antioxidant enzymes and the generation of an anti-inflammatory action to prevent oxidative stress and restore normal liver architecture after DEN toxicity. We examined the mRNA expression of AFP, IL-1β, TNF-α, and P53 to validate the development of HCC and explore the mechanism underlying the anticancer action of *Streptomyces* sp. (A16) extract. HCC is routinely diagnosed, prognosed, and screened using the tumor biomarker AFP [37]. In response to chronic liver damage or the formation of HCC, hepatic progenitor cells release AFP; increased AFP levels indicate the proliferation of these cells [38]. After injection of DEN, mRNA expression of AFP was shown to be considerably higher than in normal control mice. These increased values show the beginning of HCC in addition to liver damage. This result is in line with earlier research [39,40]. Notably, the *Streptomyces* sp. (A16) extract therapy of DEN-treated mice resulted in a decrease in AFP mRNA levels, demonstrating the anticancer effects of these therapies [41]. The presence of persistent inflammation promotes and exacerbates most types of cancer, including HCC [42].

In the current investigation, the presence of DEN as a carcinogen-induced the production of all essential proinflammatory cytokines, and this increased immune system aggression led to significant liver inflammation, as shown by the histopathological analysis and higher levels of TNF-α and IL-1β mRNA expression. The HCC was thoroughly established in this study, according to the current cytokine data and the thorough histological investigation as well. Similar findings on the harmful role of chronic inflammation in HCC have been documented in many preclinical and clinical studies [43,44]. Pro-tumorigenic inflammation, which is seen in chronic hepatitis and is characterized by the infiltration of Th2 cells, regulatory T cells (Tregs), and M2 macrophages, as well as the expression of TNF-α and IL-1β, may lead to persistent hepatocyte generation and survival, accelerating the neoplastic transformation of hepatocytes [45]. The current work demonstrates that *Streptomyces* sp. (A16) extract mediates IL-1β and TNF-α downregulation, which may contribute to the reduction in the inflammatory cascade in the DEN-treated mice. Thus, it appeared that the hepatic tissues’ improvement after being treated with *Streptomyces* sp. (A16) extract was caused by the inhibition of proinflammatory cytokines. The bioactive compounds that demonstrated anti-inflammatory effects, such as trans-Cinnamaldehyde [46] and Cinnamamide [47], may be responsible for this hepatoprotective activity of *Streptomyces* sp. (A16) extract.

The findings of this investigation demonstrate that treatment with *Streptomyces* sp. (A16) extract enhanced the histological architecture and shielded the liver from malignant histological lesions brought on by DEN. Clear hepatocellular cancerous foci, damage to the structural integrity of the hepatic lobules, necrotic liver cells with multiple inflammatory infiltrations, fatty changes (steatosis), variable nuclei sizes, loss of cell membrane, and the absence of the capillaries in the hepatic sinusoids are some of the cancerous lesions that are present in these lesions. Kupffer cell hyperplasia was also present. These pathological abnormalities are strong indications that DEN-induced hepatocarcinogenesis was a successful model.

The current study’s observation is that the levels of p53 increased after treatment with *Streptomyces* sp. (A16) extract, which is consistent with a previous study [48]. The activation of tumor cell apoptosis is one of the key mechanisms employed by chemotherapy medicines [49]. Two important pro- and anti-apoptotic proteins, Bax and Bcl-2, control the permeabilization of the mitochondrial outer membrane and the release of cytochrome C into the cytosol, which results in caspase activation and ultimately apoptosis [50]. According to our results, treatment with *Streptomyces* sp. (A16) extract up-regulated the expression of caspase-3 and Bax while down-regulating the expression of Bcl-2 in mice with DEN-induced HCC. This suggests that the regulation of *Streptomyces* sp. (A16) extract-induced hepatoma cell apoptosis may depend on increased Bax and decreased Bcl-2 expression. Studies have shown that caspase activity is essential for *Streptomyces* sp.-induced apoptosis [48]. The activation and induction of caspase-3 cleavage by *Streptomyces* sp. (A16) extract in this work suggests that this intrinsic-dependent pathway is also implicated in the mechanism of *Streptomyces* sp.-induced apoptosis. Notably, treatment with *Streptomyces* sp. (A16) triggered the apoptotic pathways in DEN-induced hepatocarcinogenesis. This was accomplished through the activation of caspase-3, up-regulation of p53 and Bax, and down-regulation of Bcl2. As a result, the primary mechanism behind the anticancer actions mediated by *Streptomyces* sp. (A16) is the activation of these common apoptotic signaling pathways. In conclusion, and according to the results of the current investigation, *Streptomyces* sp. is a significant source of bioactive substances that can be employed to treat HCC. These substances may be responsible for the recovery of antioxidant enzymes, induction of apoptosis, and production of an anti-inflammatory effect to prevent oxidative stress and restore regular liver architecture in DEN-induced hepatocarcinogenesis, which accounts for the anticancer effect of *Streptomyces* sp. extract seen in this study. Our findings support the idea that *Streptomyces* bioactive metabolites may be effective therapeutic drugs for HCC inhibition, but more research is needed to evaluate and compare the effects of these agents on human HCC xenografts. Additionally, clinical studies are needed to determine the safety and effectiveness of these drugs in human beings.

## Figures and Tables

**Table 1:** Primers used for qRT-PCR.

Gene	Forward Primer (5′-3′)	Reverse Primer (5′-3′)
AFP	CCAGGAAGTCTGTTTCACAGAAG	CAAAAGGCTCACACCAAAGAG
P53	TGAAACGCCGACCTATCCTTA	GGCACAAACACGAACCTCAAA
IL-1β	CTATGGCAACTGTCCCTGAA	GGCTTGGAAGCAATCCTT
TNF-α	GCTTGGTGGTTTGCTACGAC	ACTGAACTTCGGGGTGATTG
GAPDH	AAGGTGGAAGAATGGGAGTT	GGAAAGCTGTGGCGTGAT

**Table 2:** Phytoconstituents of ethyl acetate extract of *Streptomyces* sp. (A16) using GC-MS.

No.	Detected Compound	Formula	Retention Time (min)	Peak Area	Abundance (%)
1	Ethyl Acetate	C_4_H_8_O_2_	1.80	86,851,429	44.307
2	Propanoic acid, ethyl ester	C_5_H_10_O_2_	2.37	2,576,397	1.314
3	p-Xylene	C_8_H_10_	4.65	1,201,182	0.613
4	1,3,5-Cyclooctatriene	C_8_H_10_	5.18	345,979	0.176
5	Benzaldehyde	C_7_H_6_O	6.80	194,101	0.099
5	5-Methyl-2-furaldehyde	C_6_H_6_O_2_	6.95	321,237	0.164
6	2-Methyl-5-hexanone oxime	C_7_H_15_NO	8.15	2,498,550	1.275
7	Corylon	C_6_H_8_O_2_	8.64	230,347	0.118
8	Phenylethyl Alcohol	C_8_H_10_O	11.05	366,472	0.187
9	Larixic acid	C_6_H_6_O_3_	11.20	331,418	0.169
10	Pyranone	C_6_H_8_O_4_	11.95	150,614	0.077
11	2,4,4-Trimethyl-1-pentyl methylphosphonofluoridate	C_9_H_20_FO_2_P	13.62	460,008	0.235
12	Pyrrole-2-carboxylic acid	C_5_H_5_NO_2_	14.20	6,297,782	3.213
14	trans-Cinnamaldehyde	C_9_H_8_O	15.43	14,191,887	7.240
15	Pyrrole-2-carboxamide	C_5_H_6_N_2_O	16.59	14,191,887	7.240
16	Benzamide	C_7_H_7_NO	17.55	1,141,967	0.583
17	α-ylangene	C_15_H_24_	18.23	257,653	0.131
18	Benzeneacetamide	C_8_H_9_NO	19.12	22,818,275	11.641
19	Phenylpropanamide	C_9_H_11_NO	22.06	1,628,020	0.831
20	Cinnamamide	C_9_H_9_NO	25.23	10,206,505	5.207
21	Uric acid	C_5_H_4_N_4_O_3_	26.45	1,488,722	0.759
22	3-(Hydroxymethyl)-5-methoxyphenol	C_8_H_10_O_3_	28.65	1,955,728	0.998
23	Cyclo(leucyloprolyl)	C_11_H_18_N_2_O_2_	30.93	8,920,233	4.551
24	Chiapin B	C_19_H_26_O_6_	31.82	3,618,392	1.846
25	cis-13-Eicosenoic acid	C_20_H_38_O_2_	33.68	880,571	0.449
26	2,5-Piperazinedione, 3,6-bis(2-methylpropyl	C_12_H_22_N_2_O_2_	35.50	1,801,612	0.919
27	(Z)-9-Octadecenamide	C_18_H_35_NO	38.61	5,237,385	2.672
28	Dihydroergotamine	C_33_H_37_N_5_O_5_	38.95	4,316,002	2.202
29	cis-5,8,11,14,17-Eicosapentaenoic acid	C_20_H_30_O_2_	39.36	1,542,283	0.787

**Table 3:** Effect of *Streptomyces* sp. (A16) extracts on body weight (g), liver weights (g), and liver enzymes in DEN-administered mice.

Parameters/Groups	Control	A16 (L)	A16 (H)	DEN	DEN+ A16 (L)	DEN + A16 (H)
Body weight(g) week32	43 ± 1.6	43.6 ± 1.6	43.5 ± 3	38.8 ± 0.8 ***	39.6 ± 1.6 ^###^	38.9 ± 0.6 ^##^
Body weight(g) week36	44.4 ± 1.6	43.8 ± 1.9	44.8 ± 1.9	41.0 ± 0.9 ***	43.0 ± 2.0 ^###^	43.6 ± 0.6 ^##^
Absolute liver weight (g)	2.3 ± 0.06	2.3 ± 0.1	2.1 ± 0.1	3.3 ± 0.08 **	2.9 ± 0.08 ^##^	2.7 ± 0.08 ^###^
AST (U/L)	106.4 ± 1.11	112.6 ± 2.72	112.8 ± 0.44	227.9 ± 5.74 ***	116.7 ± 0.88 ^###^	109.0 ± 2.17 ^###^
ALT (U/L)	37.35 ± 0.5	40.51 ± 0.46	42.32 ± 0.43	125.5 ± 1.5 ***	48.76 ± 0.53 ^###^	41.00 ± 1.11 ^###^

Data are presented as mean values ± SEM and statistically analyzed by one-way ANOVA followed by Tukey–Kramer post hoc test. ** *p* < 0.01, *** *p* < 0.001 were significant compared with the control group, and ^##^ *p* < 0.01, ^###^ *p* < 0.001 were significant compared with DEN-treated mice.

## Data Availability

The data used to support the findings of this study are included within the article.
